# Students helping students: vertical peer mentoring to enhance the medical school experience

**DOI:** 10.1186/s13104-017-2498-8

**Published:** 2017-05-02

**Authors:** Christine Andre, Jessica Deerin, Luci Leykum

**Affiliations:** Division of General and Hospital Medicine, University of Texas Health Science Center San Antonio (UTHSCSA), 7703 Floyd Curl Drive, MC 7982, San Antonio, TX 78229 USA

**Keywords:** Mentoring, Peer mentoring, Career advising, Vertical peer mentoring, Medical students

## Abstract

**Background:**

Effective mentoring is an important component of medical student professional development. We provide a description of the mentoring program at our institution.

**Methods:**

Our institution UTHSCSA implemented a student-advising program (Veritas) with clinical faculty mentors and senior students (MiMs). The MiMs provided vertical peer mentoring to more junior students as an adjunct to faculty advising. The MiMs lead small group discussions that foster camaraderie, share academic and career information and promote professional identity. An optional MiM elective more intensively develops mentorship and leadership skills through a formal curriculum. The authors used annual survey data of all students as well as student mentors to evaluate program effectiveness.

**Results:**

Overall, student perception of the program improved each year across multiple domains, including feeling more prepared, supported and satisfied with their overall experience in medical school. Student mentors also found the process rewarding and helpful to their future careers as physicians.

**Conclusions:**

The authors suggest implementing a vertical peer-mentoring program can be an effective adjunct to faculty mentoring.

**Electronic supplementary material:**

The online version of this article (doi:10.1186/s13104-017-2498-8) contains supplementary material, which is available to authorized users.

## Background

Mentorship in medical school is important for career counseling, professional development, fostering interest in research, and supporting personal growth [1, 2]. Recognizing the benefits of mentorship, the Liaison Committee on Medical Education (LCME) requires schools have “an effective career advising system in place that integrates the efforts of faculty members, clerkship directors, and student affairs staff to assist medical students in choosing elective courses, evaluating career options, and applying to residency programs” [3]. LCME also requires that medical schools includes programs to promote their students well-being and to facilitate their adjustment to the physical and emotional demands of medical education [3].

To meet LCME requirements, many schools have implemented career-advising and mentoring programs [1]. Traditional faculty-based mentorship requires significant time commitments of faculty time for not only providing mentorship, but also obtaining mentorship skills [4]. Faculty time may be limited from competing commitments of clinical, teaching, and research responsibilities in increasingly constrained fiscal environments [5, 6]. Additionally, while faculty mentors may be adept at career guidance and long term planning, they may not be best positioned to address day-to-day concerns [7]. Faculty mentors may also not be equiped to give advise on studying for particular courses or succeeding in today’s updated curricula. Yet guidance on these seemingly small matters (i.e. where to buy books or study after hours) can be important to students’ daily experiences and overall wellness.

Peer or “near-peer” mentoring has the capacity to meet these types of needs. As individuals who have recently navigated similar experiences, upperclassmen are uniquely positioned to address the daily issues facing current medical students. Upperclassmen may also be perceived as more approachable for certain discussions. Faculty and upperclassmen can form a complementary alliance, each mentoring to their areas of expertise [5, 6].

Data on peer or near-peer mentoring in medical school is limited [5, 8]. We report on a vertical peer-mentoring program utilizing fourth year medical students, “Mentors in Medicine (MiMs).” We describe this program and assess its impact on student experience from mentor and mentee perspectives.

## Methods

### Program overview

**Fig. 1:**
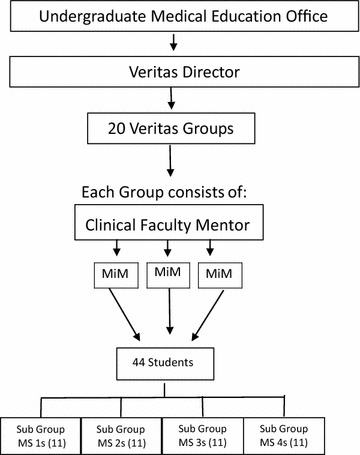
Veritas program structure

The University of Texas San Antonio School of Medicine initiated Veritas, a student-advising program, in 2006 with a goal of enhancing the professional development of medical students by fostering relationships and assisting students to make informed decisions about their careers. Approximately 220 students enter our medical school each year. At enrollment, students are randomly assigned to one of 20 Veritas groups and remain with this cohort throughout medical school. This assignment occurs with each incoming class so that across the 4 years of medical school, there are a total of 44 medical students per Veritas group (11 each of MS1-4 students). Each Veritas group has a faculty mentor (see Fig. 1).

Students receive mentorship through group and one-on-one activities. Meetings of subgroups of Veritas students (i.e., MS1s or MS2s) occur at least monthly. A subset of meetings is combined so students across classes can share experiences. Session topics are listed in Additional file 1: Appendix 1 and include: choosing a career, study strategies, professionalism, and how to plan for life as a 3rd and 4th year. There are also events for all students within a class year, such as MS4 mock-interview night or MS3 boot camp. At least twice per year, all 48 students in each Veritas group come together for a social event. Finally, Veritas-wide events such as charity drives or theme days, are sponsored.

In an effort to expand our advising capacity, we implemented a longitudinal advising system for senior students to work with rising students in 2009. Fourth-year medical students, called Mentors in Medicine (MiMs), serve as mentors to the MS1–MS4 classes. MiMs focus on navigating medical school life at UTHSCSA, choosing a specialty, obtaining extracurricular and clinical experiences, and successfully applying for residency, allowing faculty to focus their efforts on career advising and choosing a professional pathway.

**Fig. 2:**
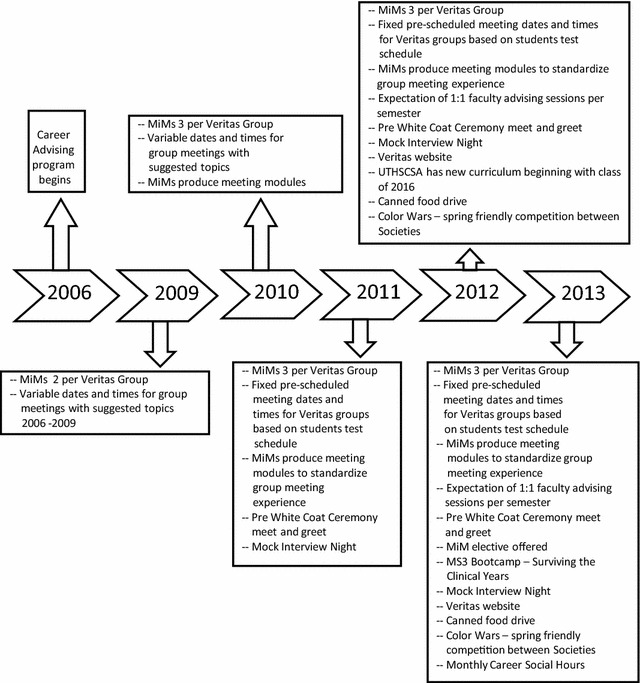
Veritas timeline and progression

Initially, two MiMs were assigned to each Veritas group. As MiM applications grew, we expanded to three MiMs per group to remain inclusive. Since 4th year students travel for interviews or away rotations, having three MiMs allows for better coverage of MiM responsibilities. While we strive for inclusiveness, the Veritas MiM program has grown in reputation, and has become competitive and prestigious. MiM applicants have grown from 40 in 2011 to 94 in the 2014–15 academic year. Figure 2 shows the timeline and progression of the Veritas program.

### Roles and responsibilities of MiMs

MiMs have primary responsibility for orchestrating group meetings, including sending reminders, running meetings, facilitating discussion, and taking notes. Faculty mentors guide MiMs in facilitating group discussions. After each meeting, MiMs submit an assessment, which is used to improve future meeting effectiveness. MiMs are also expected to make themselves available to junior students on an individual basis. MiMs participate in all milestone events such as the White Coat Ceremony.

At the year’s end, students complete a survey on their experience as a MiM, what they learned, and how they expect the experience to contribute to their future careers. Evaluation of MiM performance includes formative and summative feedback from both faculty mentors and junior students.

### Selection of MiMs

MiMs are selected from rising 3rd year students and are generally assigned to serve their own group, building on existing relationships. Students are eligible to apply if they are in good academic standing. The MiM application includes descriptions of past advising and teaching roles, and a self-assessment of mentorship skills. Faculty mentors make recommendations based on their knowledge of each applicant. Final selection is made by a committee of Veritas faculty and students, contingent upon approval by the Office of Undergraduate Medical Education. Effort was made by the committee to choose a representative sample of students with diverse backgrounds, specialty interest, traditional and non-traditional students, age and gender.

### MiM student leadership

Each year one or two students are designated MiM leaders. Students self-nominate for these positions, and are chosen based on past initiative in leading Veritas projects. Lead MiMs have a significant role in coordinating and overseeing all scheduled MiM activities. They develop meeting content under the supervision of the Veritas faculty director. Training modules, typically PowerPoint outlines, are distributed before meetings to serve as guides. These modules are updated annually. Lead MiMs also ensure MiM coverage for all group meetings, filling in as needed. Finally, Lead MiMs meet every other week with the Veritas director to share new ideas, obtain feedback and guidance on planned activities, share observations, and plan future events.

### MiM elective

In 2013, we created a yearlong, longitudinal MiM elective to complement students’ work as mentors. The objective is to more intensively develop students’ mentorship, leadership and communication skills through a formal curriculum. To meet passing requirements, students must attend a cumulative 10 h of Leadership Development. Topics include navigation of AAMC Careers in Medicine program, leading small groups, providing constructive feedback, reflection, decision-making, and authentic leadership. Sessions are recorded for students on away rotations. They must also attend at least 75% of group meetings and complete written reflections at the end of each meeting. Reflections consist of formative evaluations of group discussions, focusing on aspects that worked well and those that could be improved upon, and how their own mentoring and leadership skills played a role in the quality of their session. A more formal evaluation and self-assessment is done annually, and the results are reviewed by the Veritas director and faculty mentors.

Students must also complete a Veritas advancement and improvement project. Projects are intentionally self-directed so students develop projects based on student and program needs. Proposed projects must be approved by the Veritas Director and completed by the end of the academic year. At the completion of the project, students must present their work, including a project description, obstacles overcome, future directions, and sustainability plan. Subsequent MiMs can continue these projects. In the 2013–14 academic year, 16 projects were completed (Table 1). These projects led to several programmatic improvements, including an increase in special events, greater dissemination of career and specialty information, and increase in camaraderie between classes. Additionally, these projects have resulted in ten scholarly presentations at regional, national, and international meetings. The complete list of projects is available as Additional file 2: Appendix 2.

**Table 1:** MiM elective projects

MiM elective projects 2013–2014
Online blog highlighting details of different specialties
Comprehensive list of interest groups and volunteer activities
Wellness fair: educational session on work/life balance
Mock interview night: 4th years dress rehearsal for residency interviews with formal feedback from faculty
Third year survival guide: extensive electronic document with specific suggestions for each clerkship rotation
Post Veritas group meeting surveys: data compiled and distributed to faculty and MiMs for real time feedback and improvement
MS3 Bootcamp—surviving the clinical years: panel discussions for MS3s beginning their clinical rotations
Blackboard set up created for infrastructure of MiM elective
VSAS away rotation process presentation distributed to MS3s
MiM program oversight: 2 lead students to oversee the work of all the MiMs, communicate with faculty, create training modules
Scholarly write up: submit abstracts to regional and national meetings on MiM program
Career specialty social hour: monthly events with an opportunity for students to mingle with faculty and residents from a highlighted specialty in an informal setting
Interview of program directors for career specialty information
Financial informational session for students
Mentoring session for MS3s conducted at regional campus in Harlingen (RAHC)
Compile all MiM projects for a final presentation and to be passed on to future students

### Assessment of MiM impact

To assess the impact of the MiM initiative on the Veritas program, we used extant survey data from annual year-end Veritas evaluation surveys sent to all students. The Institutional Review Board of the University of Texas Health Science Center at San Antonio deemed our program evaluation “not human subjects research”.

End of year Veritas evaluation surveys were initially developed to assess program effectiveness and to facilitate program improvement. We sought to understand how students perceived the program and how it served their needs. The literature review on this topic was scant and no instruments were already developed to assess the impact of mentoring. Without literature to guide us, the first draft of questions was developed by program leadership and initially grounded in aspects of the Veritas program that were deemed important. These questions were reviewed by an education specialist for more optimal framing. Approximately 25 questions were sent to faculty mentors and select students for testing and feedback. The final survey was refined to 21 questions. Because the purpose of the surveys was for local assessment, no psychometric analyses were done. The survey was distributed to all students by email via Survey Monkey in the spring of 2011, 2012, 2013, and 2014. Participation was completely voluntary and anonymous. The All-Student survey has been refined over subsequent years, creating minor variations in phrasing of questions. The most recent survey is available in Additional file 3: Appendix 3. The first 10 questions are yes/no responses. The last question on satisfaction is an average of Likert scale responses from 1 to 5. The only demographic question asked related to respondent gender.

Beginning in spring 2012, we began to survey MiMs. As with the All-Student survey, our goal was to assess the MiM experience, Veritas group function, and identify areas for potential improvement. There were 16 items, with responses ranked on a 1 to 5 Likert scale. (Strongly disagree to Strongly agree, or Not at all well to Very well). The most recent survey is available in Additional file 4: Appendix 4.

For the All-Student survey we included only MSI and MSII responses, as these students were most impacted by the addition of MiMs. Questions relevant to the MiM program were compared across years. Mantel–Haenszel Chi square tests were conducted to compare differences in frequency of gender distribution in responders and medical school class and yes/no responses. Analysis of variance was conducted to compare group satisfaction means of the Likert scale data. A post hoc Scheffe correction was used to control for the family-wise error rate for alpha inflation. Data analysis was performed with SAS Software, Version 9.2. (SAS Institute. 2013. The SAS system for Windows. Release 9.2. SAS Institute, Cary, NC). Statistical tests were two-sided, and p-values of less than 0.05 were considered statistically significant.

For the MiM Survey, “Very Well” and “Somewhat well” responses were combined and compared to combined “Disagree” and “Strongly Disagree” responses. Chi square tests were conducted to compare differences in discordant responses.

For both surveys, content analysis was conducted on open-ended responses. Responses were categorized by two independent reviewers into themes. A third reviewer was used for discordant areas. All years of each survey (2012–2014) were combined because response themes were consistent.

## Results

### All-student survey

Among all MSI and MSII students, 28.9% (127/439) responded in 2011, 39.5% (183/463) in 2012, 45.0% (198/440) in 2013, and 36.8% (162/440) in 2014. There was no significant difference in the gender breakdown between survey responders and medical school class for each of the 4 years (50% females in medical school vs 59% in survey responders in 2011; 51% females in medical school vs 55% in survey responders in 2012; 46% females in medical school vs 51% in survey responders in 2013; 46% females in medical school vs 55% in survey responders in 2014). Overall, students reported significant year-to-year improvements in their Veritas experiences from 2011 to 2014 (Table 2). Post-hoc analysis revealed students’ Veritas group experiences had increased medical school satisfaction from 2011 to 2012, from 2011 to 2013, from 2011 to 2014, and from 2012 to 2014 (see Table 2).

**Table 2:** Student Veritas group perceptions

Survey questions	2011		2012		2013		2014		p value
	N	%	N	%	N	%	N	%	
Discussed professionalism questions or issues	60	48.4	130	75.6	178	91.3	150	94.3	<0.0001
Shared information regarding academic planning	94	74.6	158	91.3	183	93.9	149	93.7	<0.0001
Helped me know what to do to prepare for the next year	87	69.6	152	88.4	174	89.2	140	88.1	<0.0001
Provided networking opportunities	60	47.6	108	63.2	115	59.3	118	74.2	<0.0001
Allowed a safe place for discussion of personal issues	63	50.8	115	67.3	158	81.4	136	85.5	<0.0001
Provided peer support	94	74.6	146	84.4	180	92.8	150	94.3	<0.0001
Promoted relationships between classes	65	52.0	119	68.8	162	83.5	133	83.7	<0.0001
Helped me get to know others in my class	82	65.6	136	78.6	164	84.5	139	87.4	<0.0001
Helped me feel like I wasn’t alone	85	68.6	132	76.3	174	90.2	142	89.3	<0.0001
Discussed emotional issues related to patient care	27	22.3	77	44.8	137	70.6	128	81.0	<0.0001

	Mean	SD	Mean	SD	Mean	SD	Mean	SD	
Overall, experiences with my Veritas student group have increased my satisfaction with medical school	3.26	1.06	3.63	0.83	3.78	0.82	3.97	0.89	<0.0001

### MiM survey

MiM response rates were 70.5% (43/61) in 2012, 70.7% (41/58) in 2013, and 77.4% (48/62) in 2014. One hundred percent of MiMs in 2012 (43/43) and 2013 (41/41) and 91.7% (44/48) in 2014 said they would still volunteer to be a MiM after having served as one. Of the 4 who would not be a MiM again, 3 felt they could not fully commit because of travel for interviews and away rotations. The proportion of MiMs strongly agreeing their contributions to Veritas were valued increased from 27.9% (12/43) in 2012 to 46.3% (19/41) in 2013, to 60.4% (29/48) in 2014. According to the 2014 survey, despite no formal training in mentoring, 71% (34/48) strongly agreed they (Table 3) felt prepared to effectively lead group meetings, 85% (41/48) strongly agreed they felt prepared to provide guidance and advice to underclassmen and 81% (39/48) strongly agreed they felt prepared to mentor junior students on an individual basis. Most respondents agreed the MiM elective improved their MiM experience [77% (28/36)], helped them to be a better mentor [83% (30/36)], and enriched their leadership skills [80% (29/36)].

**Table 3:** Post-hoc test results of differences of group satisfaction for each survey year

Year comparison	Difference between means	95% confidence limits	
2014–2013	0.18907	−0.078	0.45637
2014–2012	0.33427	0.0602	0.60834***
2014–2011	0.70871	0.411	1.00642***
2013–2012	0.1452	−0.115	0.40568
2013–2011	0.51964	0.2344	0.80489***
2012–2011	0.37444	0.0828	0.66605***

*** Comparisons significant at the 0.05 level

### Open-ended responses

Most frequent responses regarding successful Veritas group needs included participation among all members (39.7%; n = 54), communication (24.3%; n = 33), and faculty member support (19.9%; n = 27). Most MiMs (61.5%; n = 96) reported enjoying the opportunity to give advice to other students and 34.6% (54/156) liked building relationships. MiMs were asked what they least liked about the program; dominant themes were time commitment/missing meetings (25.9%; n = 36), and administrative duties (20.9%; n = 29).

## Discussion

Our experiences implementing a vertical peer-mentoring program “Mentors in Medicine” within the context of our overall Veritas medical student-mentoring program has been largely successful. Our surveys of students and MiMs suggest the program has been effective from both groups’ perspectives. Adding 4th year MiMs was associated with an improvement in student perception of the mentoring program and their overall medical school experience that has been maintained over 3 years. MiM responses indicate student mentors perceived an improvement in the program, and in their own mentoring and leadership skills. This is a description of a single program at a single institution and results may not necessarily be generalizable. A limitation of our approach was that we did not conduct psychometric analyses because the purpose was for local program evaluation. However, evaluation of the gender breakdown was similar in both cohorts and is suggestive of a representative responding sample.

Our experience suggests that a peer-mentoring program can be an effective adjunct to faculty mentoring [5, 6]. Student to student mentoring can seem more authentic, as it comes from a near peer who has recently navigated the same waters. Senior students may be perceived as less intimidating and more approachable than faculty for certain matters, removing potential barriers to communication on topics perceived as sensitive [5, 7]. Peer mentoring may also lend more credibility to faculty suggestions when echoed by senior students. Timely and relevant mentoring from student mentors paired with the “big picture” wisdom that can be offered by experienced faculty creates a powerful combination providing comprehensive mentoring [5, 6].

Implementation of our vertical peer-mentoring program required relatively few resources, making it feasible for potential adoption on other campuses. We utilized the considerable energy of 4th year students, using elective credit to recognize their efforts. The opportunity to develop leadership skills and “give back” was meaningful for students, and we found them to be eager and engaged. Participation as a MiM may also have been a way for students to differentiate themselves as more competitive for residency programs. The addition of the elective allowed us to formalize the MiM experience, providing additional mentoring resources, and enriching the MiM’s leadership experience. The student mentor approach also allowed us to develop a more robust mentoring experience without a significant increase in financial costs. Our biggest barrier was the logistical difficulty of coordinating and scheduling traveling 4th year students. We overcame this by expanding the program to accommodate more senior mentors so at least one MiM or faculty mentor was available at all times. MiMs still frequently cited missing meetings as what they liked least, feeling guilty for missing meetings due to away rotations and interviews. We believe this speaks to their ownership of the program and the responsibility they feel towards their mentees.

We were surprised many MiMs felt they possessed the skills necessary for adequate mentoring at the start of the MiM program. It is unclear whether this difference is generational in that students now generally feel more comfortable giving advice, or whether their recent medical school experiences are sufficient to make them comfortable with mentoring. The number and quality of our MiM elective projects were also surprising. MiMs are in a unique position to assess their classmates’ needs, and they created several new elements that remain part of the overall program. These include mock interview nights, monthly specialty career social hour events, orientation retreat, and “how-to” guides. Many Veritas projects were amenable to scholarly submissions, allowing the unexpected benefit of providing students an early opportunity to present their work. It has also been rewarding for the faculty to facilitate the leadership development of such highly motivated students.

## Conclusions

While we report on our experiences at a single institution, providing effective student mentorship is a universal issue [1, 4, 8]. Our implementation of peer mentors utilizing benefits such as course credit and leadership skill development rather than financial resources is generally relevant. Having experienced quality mentorship, enthusiasm for our program has grown and many students desire to become involved in mentoring future classes, thus perpetuating a constantly evolving mentorship cycle. We are currently working to expand our program with the use of additional peer mentors across the MS2 and 3 years.

## Additional files

**Additional file 1: Appendix 1.** Session Topics. A bulleted list of meeting topics noting class year.

**Additional file 2: Appendix 2.** Publication and Presentations. A comprehensive listing of all previous presentations, dates, and formats.

**Additional file 3: Appendix 3.** Veritas All Student Survey. The complete survey questions as presented to students for their response.

**Additional file 4: Appendix 4.** MiM Survey. The complete survey questions as presented to MiMs for their response.

## Abbreviations

UTHSCSA: University of Texas Health Science Center San Antonio
MiMs: Mentors in Medicine (4th year medical students acting as mentors)
LCME: Liaison Committee on Medical Education
MSIs: first year medical students
MSIIs: second year medical students
