# Supplementary material for: Students helping students: vertical peer mentoring to enhance the medical school experience
Source: BMC Res Notes. 2017 May 2;10:176. doi: 10.1186/s13104-017-2498-8 (PMC5414204; doi:10.1186/s13104-017-2498-8)
Supplement: Supplementary file 1 — Additional file 1: Appendix 1. Session Topics. A bulleted list of meeting topics noting class year. [file 13104_2017_2498_MOESM1_ESM.docx]

**Appendix 1: Veritas Session Topics**

- Pre White Coat Ceremony Meet and Greet (Class Year 1)
- Goal Setting, Stress Management, Overview of Medical School, Careers in Medicine (Class Year 1)
- Reflection on Professionalism (Class Year 1)
- Q&A: everyone writes down a question or comment anonymously to generate discussion topics (Class Year 1)
- Reflection on Professionalism: critique of article on communication skills (Class Year 1)
- Reflective writing – Authentic Leadership (Class Year 1)
- Reflection on article: “Relationship, Communication, and Efficiency in the Medical Encounter” (Class Year 1)
- Wisdom Exchange, Summer Plans, Getting Involved, 3rd year schedules (Class Years 1, 2)
- Choosing Specialties, Careers in Medicine program (Class Year 2)
- Step 1 and planning 3rd year (Class Year 2)
- Professionalism and Avoiding Burnout (Class Year 2)
- Check In: Clinical Rotations and Studying for Shelf exams (Class Year 3)
- Q&A: Clinical rotations, Evaluations, Study Tips, Work-Life Balance, Specialty Choice (Class Year 3)
- Expectations as a 4th year, Planning for the Interview Trail (Class Year 4)
- Off Campus Party – with Faculty Mentor (Class Years 1, 2, 3, 4)
- Holiday Lunch – Informal Discussion on Clinical Rotations (Class Years 1, 2, 3, 4)
- End of Year Party – MiM and 4^th^ Year Send-Off (Class Years 1, 2, 3, 4)
