# Supplementary material for: Students helping students: vertical peer mentoring to enhance the medical school experience
Source: BMC Res Notes. 2017 May 2;10:176. doi: 10.1186/s13104-017-2498-8 (PMC5414204; doi:10.1186/s13104-017-2498-8)
Supplement: Supplementary file 2 — Additional file 2: Appendix 2. Publication and Presentations. A comprehensive listing of all previous presentations, dates, and formats. [file 13104_2017_2498_MOESM2_ESM.docx]

**Appendix 2: Publications and Presentations**

White B, Russell J, Andre CA, Matthews, T. “Self-Perceived Benefits of a Longitudinal Fourth-Year Mentoring Experience.” 9th Annual Innovations in Health Science Education Conference. Austin, TX. 21 Feb 2013. Poster presentation.

Andre CA, Miller W, Henzi D, Leykum LK. “Longitudinal Peer Advisors Enhance the Quality of Student Mentoring.” Southern Group on Educational Affairs/Southern Group on Student Affairs/Southern Organization of Student Representatives. Savannah, GA. 18 Apr 2013. Oral and Poster presentation.

Prasifka K, Pientka A, Andre CA, Henzi D. “New Student Retreat 101—from Development to Implementation.” Southern Group on Educational Affairs/Southern Group on Student Affairs/Southern Organization of Student Representatives. Savannah, GA. 18 Apr 2013. Oral and Poster presentation.

Navuluri N, Meyer D, White B, Andre CA, Matthews T, Henzi D. “Career Days to Increase Exposure in Medicine During Preclinical Years.” Southern Group on Educational Affairs/Southern Group on Student Affairs/Southern Organization of Student Representatives. Savannah, GA. 18 Apr 2013. Poster presentation.

White B, Andre CA, Matthews T, Henzi D. “Effectiveness of a Student-Run ‘Boot Camp’ as an Orientation to Clinical Clerkships.” Careers in Medicine Professional Development Conference. San Diego, CA. 10 Jun 2013. Poster presentation.

Greer M, Hartnett S, Andre CA. “For Students by Students: A Clerkship Survival Guide.” Association of American Medical Colleges Group on Diversity and Inclusion/Group on Student Affairs/Organization of Student Representatives National Spring Meeting. San Diego, CA. 26 Apr 2014. Poster presentation.

Davis S, Yeung K, Andre CA. “Career Social Hours Facilitate Informed Specialty Choices for Students.” Association of American Medical Colleges Group on Diversity and Inclusion/Group on Student Affairs/Organization of Student Representatives National Spring Meeting. San Diego, CA. 26 Apr 2014. Poster presentation.

Rushing J, Freed K, Andre CA. “Mock Interview Night: Developing Student Skills for the Interview Trail.” Association of American Medical Colleges Group on Diversity and Inclusion/Group on Student Affairs/Organization of Student Representatives National Spring Meeting. San Diego, CA. 26 Apr 2014. Poster presentation.

Pun S, Andre CA. “Mentoring Students in the Online Era.” 10th Annual Innovations in Health Science Education Conference. Austin, TX. Demonstration.

Surapaneni K, Meyer D, Navuluri N, Andre CA. “Student Mentoring Elective.” 18th Annual International Association of Medical Science Educators Meeting. Nashville, TN. 7 Jun 2014. Oral and poster presentation.
