# Supplementary material for: Students helping students: vertical peer mentoring to enhance the medical school experience
Source: BMC Res Notes. 2017 May 2;10:176. doi: 10.1186/s13104-017-2498-8 (PMC5414204; doi:10.1186/s13104-017-2498-8)
Supplement: Supplementary file 3 — Additional file 3: Appendix 3. Veritas All Student Survey. The complete survey questions as presented to students for their response. [file 13104_2017_2498_MOESM3_ESM.docx]

Veritas All Student Survey

Thank you for helping us evaluate the effectiveness of Veritas. The survey should take no longer than five minutes to complete. Please answer all questions based on your personal experience with your Veritas faculty mentor and student group during 2013-2014 the academic year only. Your responses will be confidential—we have not asked for your name or any other personally identifying data.

1. Who was your faculty mentor:
2. Faculty mentors can serve a number of different roles or functions. Please indicate how much your Veritas Faculty Mentor assisted you with these tasks:

Strongly Agree – Agree – Neutral – Disagree – Strongly Disagree – N/A

Provided career guidance and help towards choosing a specialty

Planned end of 1^st^ year break

Planned 3^rd^/4^th^ year rotations

Provided emotional support when needed

Took time to get to know me

Helped me manage stress when needed

Helped me find additional mentors within my specialty of interest

1. Please answer these questions about your relationship with your Veritas Faculty Mentor:

Strongly Agree – Agree – Neutral – Disagree – Strongly Disagree – N/A

My mentor was a role model for me

My mentor seemed unsure of his/her role

My mentor was approachable

I would have liked to get to know my mentor better

My mentor made him/herself available to me

MIMS

1. Fourth year student mentors (MiMs) can serve a number of different roles or functions. Please indicate how much your MiM assisted you with these tasks:

Strongly Agree – Agree – Neutral – Disagree – Strongly Disagree – N/A

Took time to get to know me

Planned 3^rd^/4^th^ year rotations

Helped me manage stress when needed

Provided guidance and advice for the clinical years

Provided career guidance and help towards choosing a specialty

Provided emotional support when needed

1. For some issues or questions, I felt more comfortable talking to my MiM than my faculty mentor.

Strongly Agree – Agree – Neutral – Disagree – Strongly Disagree – N/A

1. MiMs were a useful resource and important contribution to the Veritas program.

Strongly Agree – Agree – Neutral – Disagree – Strongly Disagree – N/A

VPAs

1. Second year mentors (VPAs) can serve a number of different roles or functions. Please indicate how much your VPAs assisted you with these tasks:

Strongly Agree – Agree – Neutral – Disagree – Strongly Disagree – N/A

Took time to get to know me

Helped me understand the ins and outs of medical school

Helped me manage stress when needed

Provided emotional support when needed

Made my medical school experience more fun

1. For some issues or questions, I felt more comfortable talking to my VPA than my MiM or Faculty Mentor.

Strongly Agree – Agree – Neutral – Disagree – Strongly Disagree – N/A

1. Second year student mentors (VPAs) were a useful and important contribution to the Veritas program.

Strongly Agree – Agree – Neutral – Disagree – Strongly Disagree – N/A

1. In the last academic year (2013-2014), how many times have you had contact with the following people, including all forms of communication (in person, by telephone, email, text, etc.) excluding contact as an attending or lecturer?

Never – Rarely – Sometimes – Frequently – Always – N/A

Faculty Mentor

Veritas Peer Advisor (VPA)

Mentor in Medicine (MiM)

Your Veritas Group

1. Your Veritas GROUP may have provided a number of different functions or opportunities. Please answer these questions based upon your personal experience with your Veritas GROUP.

Yes – No

Discussed professionalism questions or issues
Shared information regarding academic planning

Helped me know what to do to prepare for the next year

Provided networking opportunities

Allowed a safe place for discussion of personal issues

Provided peer support

Promoted relationships between classes

Helped me get to know others in my class

Helped me feel like I wasn’t alone

Discussed emotional issues related to patient care

Helped me understand the ins and outs of medical school

Helped me plan what to do with break between MSI and MSII year

Developed a strategy for taking USMLE Step I exam

Planned 3^rd^/4^th^ year rotations

Provided career advising and helped me choose a specialty

Provided emotional support when needed

Discussed stress management

Helped me with work/life balance issues

1. Overall, I have been satisfied with the level of support I received through the Veritas program.

Strongly Agree – Agree – Neutral – Disagree – Strongly Disagree – N/A

1. In the last academic year (2013-2014), how many times did you attend your Veritas student GROUP meetings?

Never – Less than half – About half – More than half – All

1. The amount of time I spent meeting with my Veritas student GROUP was:

Far too little – Too little – About right – Too much – Far too much

1. Regarding the content of your group meetings:

Strongly Agree – Agree – Neutral – Disagree – Strongly Disagree – N/A

I enjoyed the topics

There was an appropriate balance of information content and room for discussion

Meetings should be more structured

Group size was about right

I wish there were more meetings with members of other class years

Optional additional comments:

1. Overall, experiences with my Veritas student GROUP have increased my satisfaction with medical school.

Strongly Agree – Agree – Neutral – Disagree – Strongly Disagree – N/A

1. Veritas is a beneficial and important part of medical school land should be continued.

Strongly Agree – Agree – Neutral – Disagree – Strongly Disagree – N/A

1. For the last academic year (2013-2014), which class of medical school were you?

MSI – MSII – MSIII – MSIV

1. Please use the space below to make additional comments or recommendations on how Veritas could be improved.
2. Do you have any other comments on your experience with the Veritas?

Thank you!
