# Supplementary material for: Students helping students: vertical peer mentoring to enhance the medical school experience
Source: BMC Res Notes. 2017 May 2;10:176. doi: 10.1186/s13104-017-2498-8 (PMC5414204; doi:10.1186/s13104-017-2498-8)
Supplement: Supplementary file 4 — Additional file 4: Appendix 4. MiM Survey. The complete survey questions as presented to MiMs for their response. [file 13104_2017_2498_MOESM4_ESM.docx]

Veritas MiM End of Year Survey

Your answers are completely confidential and are intended to improve the quality of the program. We greatly appreciate your contributions and your thoughtful answers to this survey!

1. Who was your Faculty Mentor?
2. As a MiM, how much do you agree with the following statements? I felt prepared to:

Strongly Agree – Agree – Neutral – Disagree – Strongly Disagree

Effectively lead the group meetings

Provide guidance and advice to underclassmen

Mentor students on an individual basis

Know where and when to refer underclassmen to other resources

Serve as a liaison between my faculty mentor and the underclassmen

I felt my contributions to Veritas were valued

1. What do you think are the most important characteristics of a MiM?
2. How often did you engage in the following activities as a MiM?

Never – Rarely – Sometimes – Frequently – Very Frequently

Communicated with my faculty mentor (outside of group meetings)

Communicated with my fellow MiMs (outside of group meetings)

Communicated with the underclassmen assigned to me (outside of group meetings)

Attend the scheduled Veritas group meetings

Provide guidance and advice to underclassmen on an individual basis

Learn about leadership and mentoring skills

Develop and practice leadership skills

Administrative work – meeting reminders, meeting minutes, participating in surveys

Organized or participated in other events with my Veritas group

1. How well do you think your Veritas Group did on the following?

Not at all well – Not that well – Neutral – Somewhat well – Very well

Helped students feel like they weren’t alone

Helped students understand the ins and outs of medical school

Shared information regarding academic planning

Helped students plan what to do with break between MSI and MSII year

Developed a strategy for taking USMLE Step I exam

Helped students know what to do to prepare for the following year

Planned 3^rd^ year rotations

Helped students choose a specialty

Discussed professionalism questions or issues

Provided networking opportunities

Provided emotional support when needed

Promoted relationships between classes

Helped students get to know others in their own class

Discussed emotional issues related to patient care

Discussed stress management

Allowed a safe place for discussion of personal issues

Helped students with work/life balance issues

Faculty mentor took time to get to know the students

Faculty mentor was knowledgeable

Faculty mentor gave good advice to the Group

1. What do you think it takes to have a successful Veritas group?
2. Please rate the usefulness of the following resources provided:

Very helpful – Somewhat helpful – Neutral – Not very helpful – Not at all helpful – N/A

Modules sent by email before the meetings

Careers in Medicine website by AAMC

Veritas Website

1. What additional resources do you think would have been helpful to you as a MiM?
2. What topics of interest have come up in your Veritas Group discussions that are not currently covered sufficiently? Are there additional topics you think we should be covering?
3. Do you think any of your experiences as a MiM will be useful to you in your residency years and beyond? If yes, please explain.
4. How do you think the MiM and VPA roles can best complement each other?
5. What do you like most about being a MiM?
6. What do you like least about being a MiM?
7. Knowing what you know now, would you still volunteer to be a MiM?

Yes – No why?

1. Did you complete the Veritas Mentors in Medicine Longitudinal Elective?
2. If you completed the Veritas Mentors in Medicine Longitudinal Elective, please rate your experience from the course:

Strongly Agree – Agree – Neutral – Disagree – Strongly Disagree

The elective course improved my MiM experience.

The elective course helped me be a better mentor.

The elective course enriched my leadership skills.

The elective course helped me enhance my teaching skills.

The elective course improved my presentation skills.

The elective course enabled me to work on a specific project.

The elective course will enrich my curriculum vitae.

Please list any other way the MiM Elective had an impact on your medical education:

1. Any other parting comments?

Thank you!
